# The value of ICG-guided left colon vascular variation and anatomical rules for the radical resection of proctosigmoid colon cancer

**DOI:** 10.3389/fonc.2023.1259912

**Published:** 2023-11-03

**Authors:** Jiahao Wang, Jiaxin Xie, Xinquan Lu, Jiaxin Lin, Weilin Liao, Xiaojiang Yi, Xiaochuang Feng, Bosen Zhu, Wenjuan Li, Xin Tang, Lin Ao, Zhifeng Chen, Hongming Li, Dechang Diao

**Affiliations:** ^1^ The Second Clinical Medical College, Guangzhou University of Chinese Medicine, Guangzhou, China; ^2^ The Second Affiliated Hospital of Guangzhou University of Chinese Medicine (Guangdong Provincial Hospital of Chinese Medicine), Guangzhou, China

**Keywords:** proctosigmoid cancer, indocyanine green, marginal artery, vascular variation, measurement

## Abstract

**Objective:**

During laparoscopic radical resection for proctosigmoid colon cancer (PCC), surgeons could inadvertently damage the arteries when following the operation path.This study investigated the variations in left colon blood vessels in order to guide the scientific protection of the marginal artery (MA) during laparoscopic surgery for PCC.

**Methods:**

Data from seven patients who underwent inferior mesenteric artery (IMA) angiography were included as imaging references to preliminarily explore the vascular structure and variation in the left colon. The clinical video data of 183 PCC patients were retrospectively analyzed to observe intraoperative MA injury. Meanwhile, a prospective cohort of 96 patients with the same disease underwent intraoperative indocyanine green (ICG) fluorescence imaging of the peripheral sigmoid artery network, the variation of marginal arteries was summarized, and the distance between vessels and the bowel was measured at different levels. Patients were divided into ‘ICG group’ and ‘non-ICG group’ according to whether ICG guidance was performed, and perioperative conditions were compared between the two groups. Taking the integrity of lymph node dissection into consideration, 18 patients underwent carbon nanonode tracing. This study was conducted under the standard consent and ethical approval of the Ethics Committee of our center.

**Results:**

7 patients with IMA angiography shared some vascular structures, defined as ‘Dangerous Triangle’ and ‘Secure Window’. Through intraoperative observation, the primary arch was typically located 4.2 (2.3-6.0) cm away from the intestinal canal, and 5.21% (5/96) patients had poor anastomosis at the primary arch. Moreover, secondary vascular arches (6.4 (4.6-10.0) cm from the intestinal wall) were observed in 38.54% of patients. MA injury was identified in 2 of 183 cases, and the ischemic bowel was timely dissected, whereas no such injury occurred during ICG fluorescenceguided surgery. Guided by carbon nanoparticles, the integrity of lymph node dissection can be maintained while preserving the secondary arch in all patients.

**Conclusions:**

This study demonstrated the benefits of ICG guidance in protecting the intestinal blood supply in laparoscopic PCC surgery. By enhancing the understanding of primary and secondary vascular arches, secure windows, and dangerous triangles, surgeons can safely optimize the surgical path during surgery.

## Introduction

The colonic marginal artery (MA) is a tortuous and slender blood vessel that runs along with the colonic bowel and directly supplies blood to the intestine (1); hence its integrity and patency are essential for intestinal survival (2). The variation in mesenteric vasculature influences the course of marginal arteries. However, the vascular architecture of the left-side colon remains a controversial topic, especially for the MA. Meanwhile, the intraoperative protection mode of MA remains unknown. Due to technical limitations, the MA has only been detected in 25% of patients (3). Currently, relevant studies on the presence of MA are limited.

Nowadays, complete mesocolic excision (CME) and central vascular ligation (CVL) are the mainstream concepts of sigmoid and rectal colon cancer surgeries (4). The mesentery is mobilized along the level of the IMA root to maximize the efficiency of lymph node dissection. The left colonic artery (LCA) and the inferior mesenteric vein (IMV) are also severed at this level. The MA might be transected during CVL and mesentery mobilizing. Normally, this would be compensated by the formation of collateral circulation. However, if the marginal arch is absent or poorly anastomosed, the likelihood of intestinal ischemia increases.

Previously, the most reliable technique to protect the blood supply was to visualize or feel the pulsation of the arch during the operation, but it was challenging to recognize it via a laparoscope, especially in patients with obesity or mesenteric hypertrophy. Surgeons must meticulously separate the mesentery and the arteries. It has been reported that the MA is located approximately 2.5 cm away from the colon (1). However, the intestinal wall and the MA are not parallel. The data has minimal clinical significance for surgical precision, and surgeons could inadvertently damage the arch when following the operation path, resulting in more intestines to be resected (5), additional operation time (having to mobilize the spleen flexure), and an increased risk of postoperative anastomotic leakage (6).

Besides, the inherent weakness and variation of the MA may also result in postoperative intestinal ischemia, depending on the expertise of surgeons. During the course of the MA, poor anastomosis or insufficient perfusion may exist. The three most common weaknesses are (1): the anastomosis of the ileocolic artery with the right colonic artery (2); Griffiths’ critical point, the anastomosis of the left colonic artery and middle colonic artery (3); Sudeck’s point, the anastomosis of the inferior sigmoid artery and the superior rectal artery. When the blood supply of the marginal arch is insufficient, it is mostly compensated by the collateral circulation or secondary arch (such as the Riolan arch, the branches of the LCA, and the sigmoid artery). Opinions vary when it comes to the vascular variation of the left colon. Many scholars have proposed reasonable classifications of left colon vascular variation, including Testut, Latarjet, Zebrowski, YaDa, Predescu, Murono, etc (7–9). These classifications guide us to correctly identify the source of intestinal blood supply; however, they cannot assist in effectively avoiding vascular injury.

Herein, we attempted to find a feasible method to protect marginal vessels based on previous left colon vascular variations. The distance between the marginal artery and the bowel was measured, and the development of the primary arch and the secondary arch branching from the sigmoid artery was observed. This research aimed to scientifically limit the number of limbic artery injuries in order to reduce unnecessary related postoperative complications. This study may be helpful to ensure both the quality of lymph node dissection and the integrity of intestinal blood circulation during CME surgery for sigmoid colon cancer and has major reference and application value for standardization and precision.

## Method

### IMA angiography

Our team retrieved part of the images of the previous IMA angiography performed in the interventional department of our center. The course and anastomoses of the marginal artery were angiographically observed in these patients.

### Previous surgical peripheral artery injury

A total of 183 patients with colorectal cancer diagnosed in our center from January 2020 to August 2021were retrospectively enrolled. The medical records of hospitalization and matched surgical video were reviewed, and marginal artery injury during surgery was observed.

### Observation of marginal artery by ICG

From December 2020 to December 2021, 96 patients with a confirmed diagnosis of colorectal cancer underwent ICG-guided radical resection of colorectal cancer. Patients with a history of abdominal surgery and ICG allergy were excluded, and informed consent was obtained. All patients underwent conventional abdominal exploration. 25 mg ICG was dissolved in 10 mL normal saline in advance, and then 6 ml was injected through the central venous catheter. Under the guidance of endoscopic fluorescence, the location of blood vessels and primary and secondary arches at the edge of the colon was determined. The bleeding pipe network was outlined with methylene blue dye. Next, the type of IMA vascular variation was recorded. The horizontal distance between blood vessels at all levels and the intestinal wall was measured with a ruler. All patients underwent preoperative carbon nanoparticle lymph node tracing to ensure the integrity of lymph node dissection.

### Measuring approach

**Figure 1:**
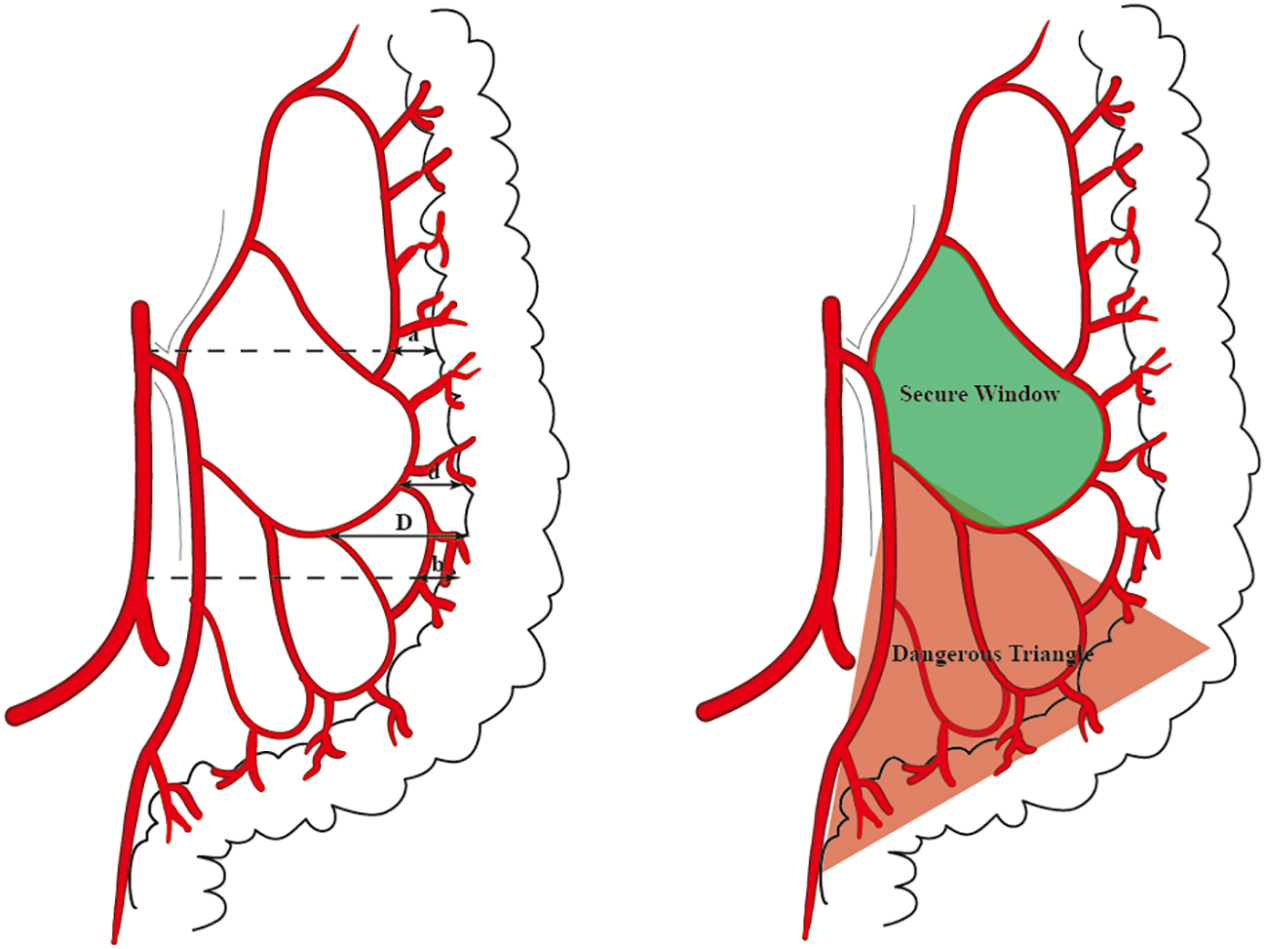
The distance that we measured at four levels is marked by arrows **(A)**. The ‘Secure Window’ is painted with blue and the ‘Dangerous Triangle’ is outlined in red **(B)**.

The distance between the medial wall of the marginal artery and the bowel was measured at four levels: (1) the level of the IMA root(a), the first marginal artery to be touched when the mesentery is cut horizontally during high ligation;( 2) the branch level of the iliac artery(b);( 3) the primary arch(d);(4) the secondary arch(d) (**Figure 1**). When the MA develops poorly in patients with mesenteric hypertrophy, the distance was measured ex vivo.

### Comparative analysis

The patients included above were divided into two groups: the non-ICG group (direct radical resection of colon cancer without ICG developing blood vessels) and the ICG group (direct radical resection of colon cancer with ICG guidance). The baseline data of all patients were collected, including age and gender, tumor location, and differentiation degree. The perioperative conditions of patients were tallied, including operation time, intraoperative blood loss, postoperative hospitalization time, total hospitalization time, pathological staging of the tumor, and postoperative complications. Statistical methods were used to compare the differences in baseline information and prognosis between the two groups.

### Nanocarbon lymphatic tracer

18 PCC patients were prospectively included in this study. They underwent endoscopic submucosal injection of carbon nanoparticles one day prior to the surgery. Therefore, researchers could observe the relationship between deep-stained lymph nodes and the surgical path during the operation.

This study is conducted under the standard consent and ethical approval of Ethics Committee of our center (ZF2020-328).

### Statistical analysis

The intraoperative measurement data were transferred to the computer, and the statistical analysis was performed by SPSS 26.0 data analysis program. The data summary is expressed as mean ± standard deviation, percentage and frequency. The parameter values were tested by Fisher exact probability method or χ2 test, and the continuous values were tested by Student t test. P < 0.050 was considered significant.

## Result

### IMA angiography

**Figure 2:**
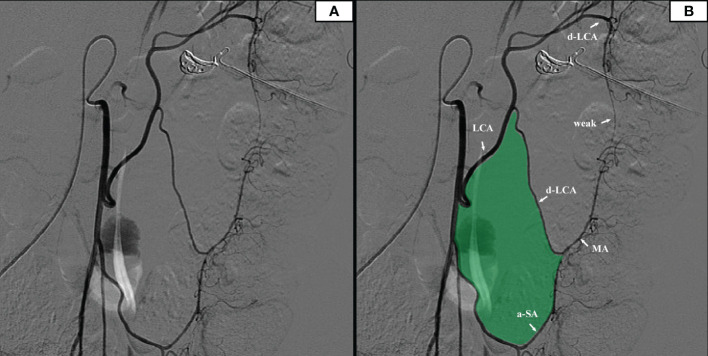
**(A)** The original image of the IMA angiography. **(B)** IMA angiography showed that there was a relatively open vascular-free area on the left side of the left colon artery (filled with green).

7 patients underwent IMA angiography in the interventional department of our center from January 2018 to December 2021. By comparing the blood vessels of IMA in these patients, some common structures were identified. There was a vascular-free zone on the left side of LCA in these patients, usually larger than 3 cm in diameter. Under the guidance of CME, this area can be easily penetrated. It is more convenient to dissociate IMV and LCA, which is very helpful in guiding mesangial clipping and represents a priority breakthrough window for the anatomical dissection of IMV and LCA. This window was labeled as ‘Secure Window’. Its four boundaries are: LCA descending branch (upper bound), ascending branch of the first sigmoid artery (lower bound), LCA (inner bound), and descending colonic marginal artery (lateral). The vascular structure is often complex in the area below the first sigmoid artery; there are many SA and its anastomosis of the vascular arch. This area was coined the ‘Dangerous Triangle’. In this area, the secondary arch of the edge arch may co-exist, or it may be absent. When the edge arch anastomosis is adequate, the secondary arch is markedly weak. When the edge arch anastomosis is poor, the secondary arch is compensated, and then it is often more than 5 cm away from the intestinal wall. At this time, it is easy to damage the edge artery arch according to the 3 cm standard. Its boundaries are: the first sigmoid artery (upper bound), IMA distal (inner bound), and sigmoid marginal artery (lateral bound) (**Figure 2**).

### Previous surgical peripheral artery injury

**Figure 3:**
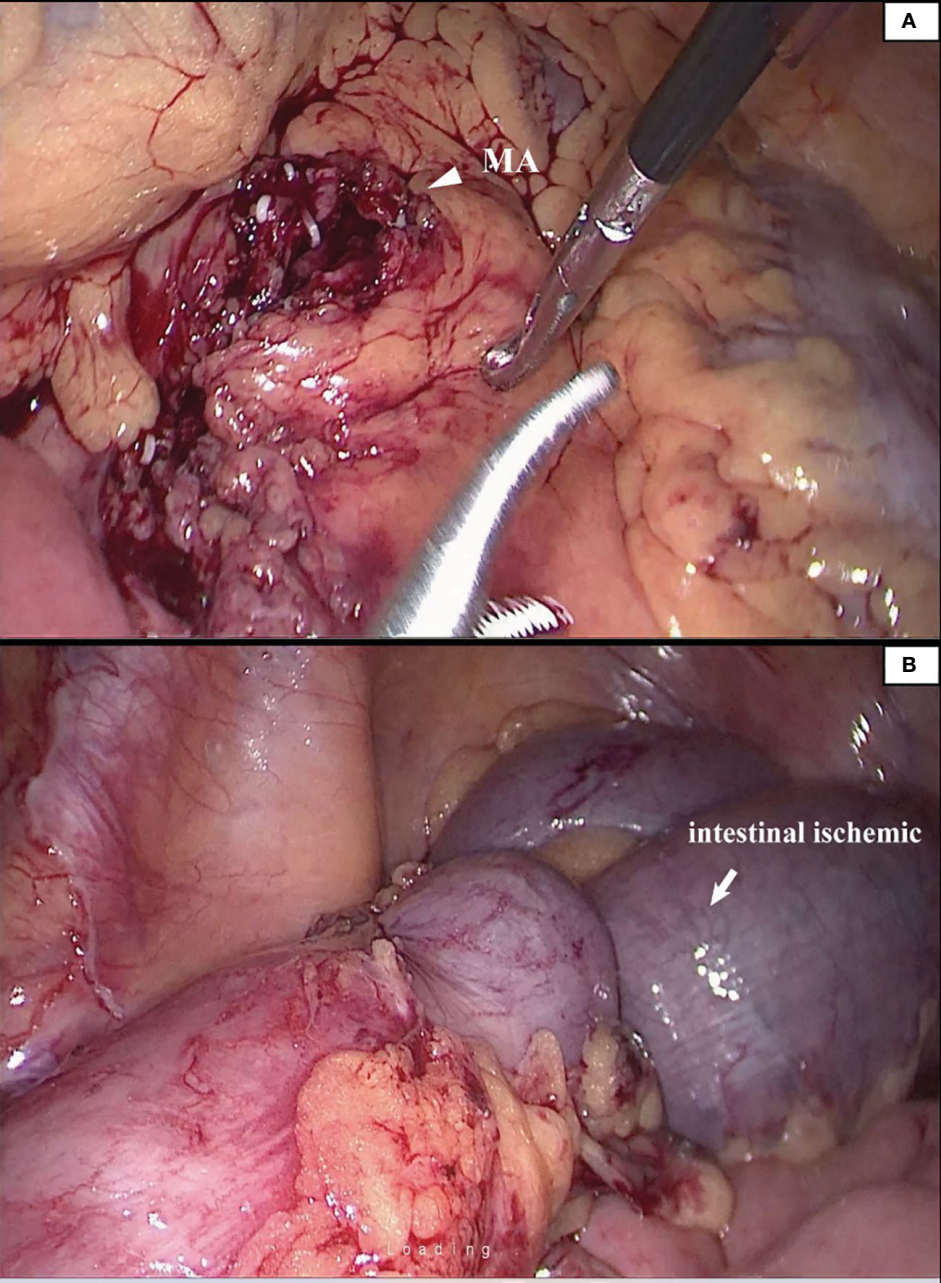
The patient suffered from mesenteric hypertrophy **(A)**. During the operation, the blood vessel arch at the edge of the lesion was damaged to the ischemia of the intestinal canal **(B)**. The operator timely detected and ruptured the necrotic intestinal canal.

We reviewed the medical records of 183 patients who underwent radical resection of colorectal cancer and matched the surgical video database. We found that there were 2 cases of intraoperative marginal artery injury. The two patients were found with intestinal ischemia, and the necrotic intestinal canal was ruptured (**Figure 3**).

### Observation of the marginal artery

**Figure 4:**
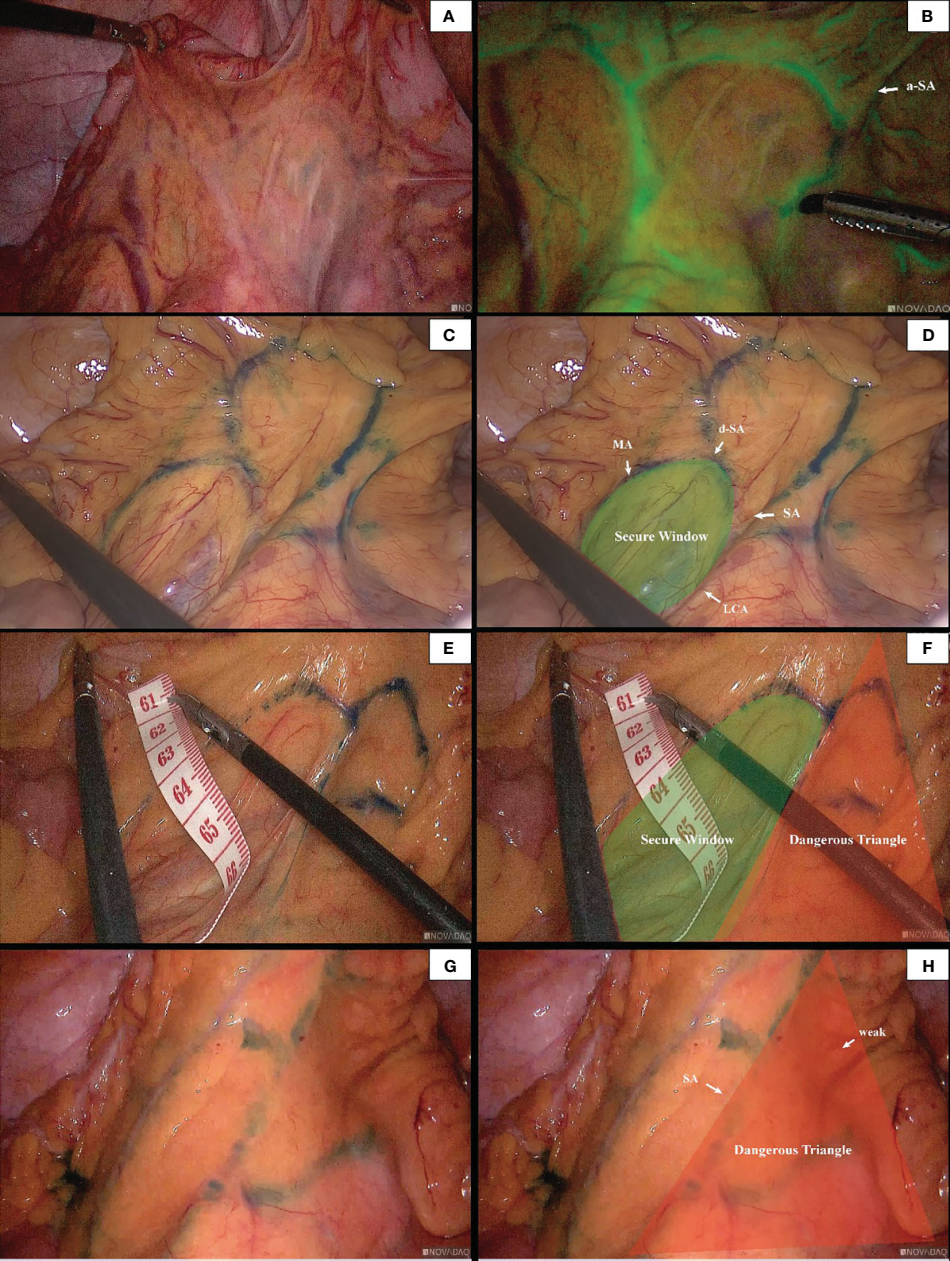
The anastomosis of vascular network can be observed under ICG-guided laparoscopic surgery **(A, B)**. We used methylene blue dye to outline the anastomosis form of marginal vascular network **(C, D)**, and observed the existence of ‘Secure Windows’ **(E, F)** and ‘Dangerous Triangles’ **(G, H)**.

**Figure 5:**
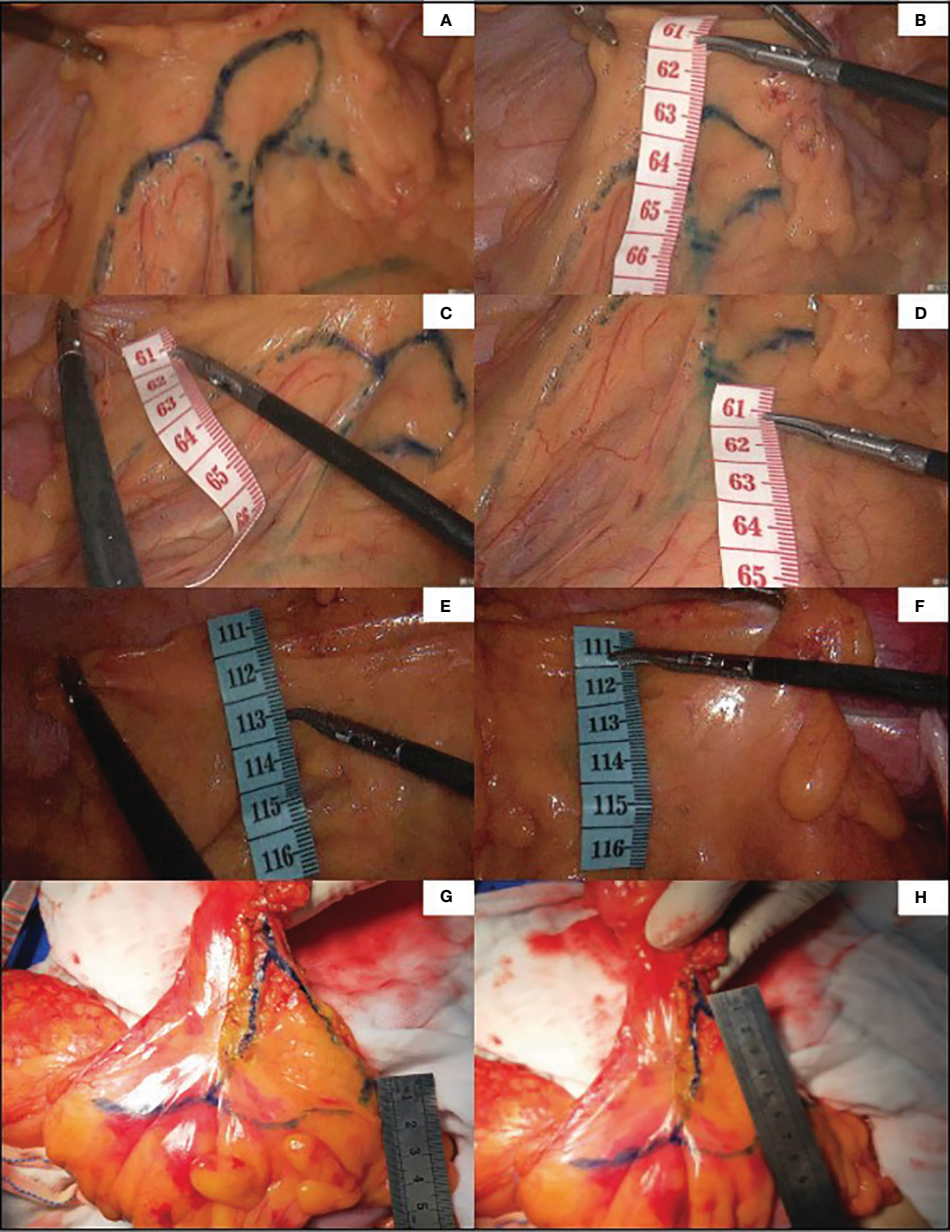
Guided by ICG, the marginal vascular network was outlined with methylene blue dye **(A)** and the distance from the marginal artery to the intestinal wall was measured. **(B)** is the distance between primary arch and intestinal wall, **(C)** is the level of IMA root, **(D)** is the distance between secondary arch and intestinal wall. **(E, F)** demonstrate the difficulty of imaging marginal vessels in patients with mesenteric hypertrophy.In vitro measurement of ICG angiography in patients with mesenteric hypertrophy **(G, H)**.

In 96 cases of radical resection of colorectal cancer guided by ICG angiography, 82 had clear ICG fluorescence, whereas 14 cases had poor imaging. The anastomosis of the vascular network was observed during the operation (**Figure 4**). The safety windows and dangerous triangles could be observed as well. Guided by ICG angiography, the location of the marginal vessels and primary and secondary arches of the colon were visualized, and their course was outlined with methylene blue dye. The horizontal distance between blood vessels at all levels and the intestinal wall was measured with a ruler (**Figure 5**)

Descriptive measurements determined that the Drummond marginal artery adjacent to the bowel was detected in all patients. The marginal artery was 3.02 ± 0.68 cm from the intestinal wall at the root level of IMA and 3.12 ± 0.74 cm from the intestinal wall at the branch level of the iliac artery. Moreover, the distal end of the marginal artery (primary arch) was 4.16 ± 0.75 cm from the intestinal wall. On the other hand, the secondary arch was identified in 37 patients (38.54%), and the distance from the secondary arch to the intestinal wall was 6.79 ± 1.39 cm (**Table 1**).

**Table 1:** The horizontal distance between blood vessels at all levels and intestinal wall, the proportion of blood vessel arch, and the position relationship of inferior mesenteric vein with LCA and marginal artery at IMA level are shown above.

Items	Number of patients(N=96)	Distance(cm)	Proportion
**Primary arch(d)**	96	4.2(2.3-6.0)	100%
**Secondary arch(D)**	37	6.4(4.6-10.0)	38.54%
Marginal artery			
IMA root(a)	96	3.0(1.2-4.6)	100%
Branch level of the iliac artery(b)	96	3.0(1.6-4.5)	100%
IMV&LCA			
Type A	21	/	21.9%
Type B	46	/	47.9%
Type C	28	/	29.2%
IMV absence	1	/	1.0%
IMV&MA			
Close-type	1	/	1.0%
Loose-type	94	/	97.9%

**Figure 6:**
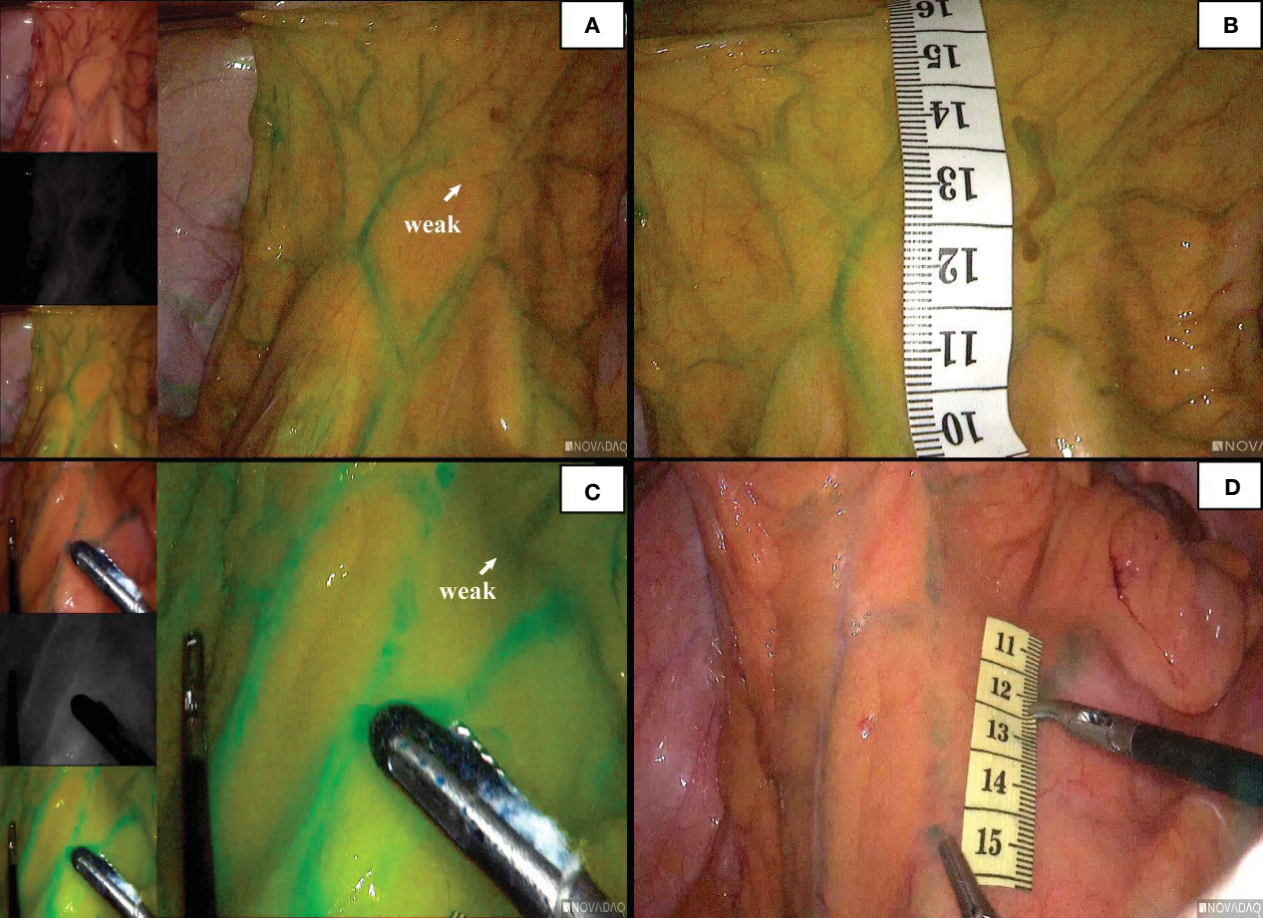
The patient ‘ s marginal arch shown in the figure is poorly anastomosed **(A)**. At this time, the blood supply was compensated by the secondary arch **(C)**. The distance between the secondary arch and the intestinal wall was measured **(B, D)**.

Poor marginal arch anastomosis was identified in 5 patients (5.2%), with the secondary arch compensating for blood flow (**Figure 6**).

### The relationship between the inferior mesenteric vein with LCA and marginal artery at the IMA level

**Figure 7:**
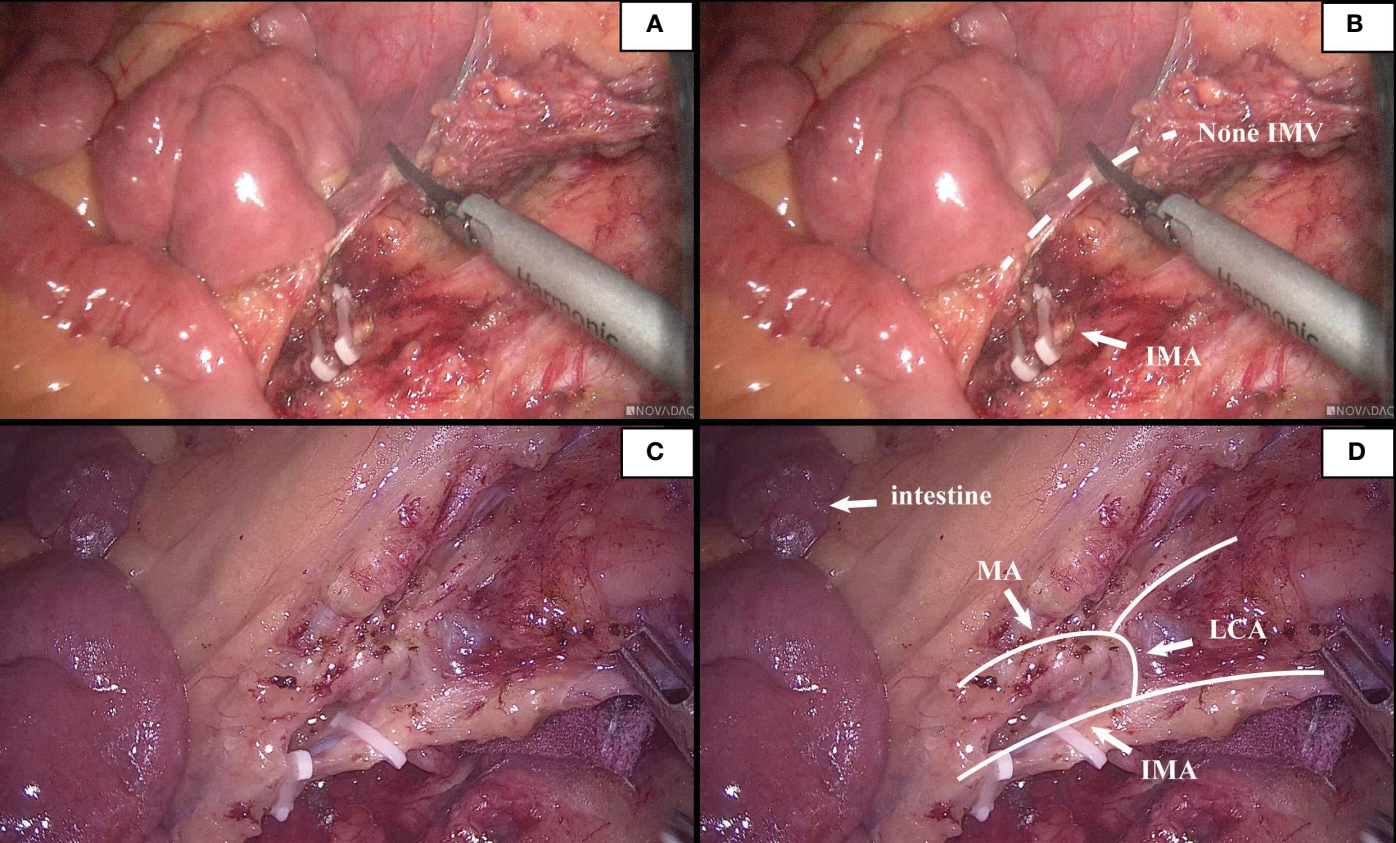
**(A, B)**. This patient had poor intestinal rotation, and his IMV was absent. **(C, D)**. A patient had poor intestinal rotation, his ascending branch of the LCA forms directly part of the marginal arch, secure window could not be seen.

According to the characteristics of IMV in the root plane of IMA by the Japanese scholar Munoro (9), the relationship between IMV and nearby blood vessels was observed and classified. It was found that the location relationship between IMV and LCA belonged to type A in 21 cases (21.9%), type B in 46 cases (47.9%), and type C in 28 cases (29.2%). At the root level of IMA, IMV and the marginal artery were usually distinct (94/96,97.9%). In this study, 2 patients with poor intestinal rotation were identified: one patient with IMV deficiency (**Figure 7**); in the second case, the IMV was close to the MA, and the ascending branch of the LCA directly formed the marginal artery; no secure window was visualized.

### Comparative analysis

There was no statistically significant difference in age, gender, BMI, pathological type and staging between the ICG group and the non-ICG group. Intraoperative blood loss in the ICG group was 27.74 ± 24.07 mL, which was statistically significant compared with 75.73 ± 141.99 mL in the non-ICG group (p < 0.05). However, there was no significant difference in the operation time (185.62 ± 53.57 min vs. 193.48 ± 63.97 min), total hospitalization time (11.54 ± 3.09 vs. 11.85 ± 4.59 d), and postoperative hospitalization time (5.88 ± 2.00 d vs. 6.79 ± 3.10 d) between the ICG group and the non-ICG group (**Table 2**). Intraoperative condition: No vascular injury or marginal artery injury occurred in the ICG group. In the non-ICG group, there were 2 cases of intraoperative injury of the marginal artery and 4 cases of vascular injury. Postoperative complications: 1 case of postoperative intestinal obstruction (grade IIIb according to Caliven-Dindo system); postoperative anastomotic leakage occurred in 1 case (grade II), and the patient was treated conservatively; postoperative cardiac insufficiency (grade II) developed in 1 case. There were 2 cases of marginal artery injury in the non-ICG group (**Figure 1**); 1 case of postoperative cardiac insufficiency (IV), and the patient was monitored in the ICU; 5 cases of postoperative anastomotic leakage (IIIb), and the patients were treated surgically; one case of venous thrombosis of the lower extremity (grade IIIa); mild complications (grade I ~ II) included 1 case of anastomotic leakage, 1 case of hypovolemic shock, 1 case of intestinal obstruction, 2 cases of pulmonary infection, and 1 case of lymphatic leakage. Collectively, the aforementioned findings demonstrate that ICG-guided radical resection of colorectal cancer can minimize intraoperative blood loss and perioperative complications (**Table 3**). All patients underwent preoperative carbon nanoparticle lymph node tracing to ensure the integrity of lymph node dissection while preserving intestinal blood supply.

**Table 2:** Baseline information of patients.

Variables	Number of patients ICG group(N=82)	Number of patients No ICG group(N=183)	P value
Age (years)			
Median **±** SD	62.79 **±** 12.45	64.31 **±** 12.94	0.376
BMI (kg/m^2^)			
Median **±** SD	22.03 **±** 2.88	21.00 **±** 2.8	0.564
Sex			
Male	51	119	0.378
Female	31	64	
Tumor differentiation			
Poorly differentiation	4	96	0.565
Moderately differentiation	64	12	
Mucous adenocarcinoma	3	6	
Others	12	6	
**Carcinogenesis **Yes	74	168	0.338
** **No	8	15	
TNM stage			
Tis	2	13	0.459
I	12	19	
II	28	44	
III	29	40	
IV	4	4	
**Surgical time(min)**	185.62 **±** 53.57	193.48 **±** 63.97	0.332
**Intraoperative blood loss(ml)**	27.74 **±** 24.07	75.73 **±** 141.99	<0.05
**Inpatient days**	11.54 **±** 3.09	11.85 **±** 4.59	0.514
**Postoperative inpatient days**	5.88 **±** 2.00	6.79 **±** 3.10	0.413
Postoperative complications			
Yes	3	13	0.213
No	79	170	
Classification of complications (Caliven-Dindo system)			
I	0	1	0.886
II	2	5	
IIIa	0	1	
IIIb	1	5	
IV	0	1	

**Table 3:** Perioperative complications.

Complications	Number of patients ICG group(N=82)	Number of patients No ICG group (N=183)
Intraoperative		
Marginal artery injury	0	2
Vascular injury	0	4
Postoperative		
Anastomotic leakage	1	6
Lung infection	0	2
Chylous leakage	0	1
Ileus	1	1
Cardiac insufficiency	1	1
Hypovolemic shock	0	1
Venous thrombosis	0	1

### Nanocarbon lymphatic tracer

**Figure 8:**
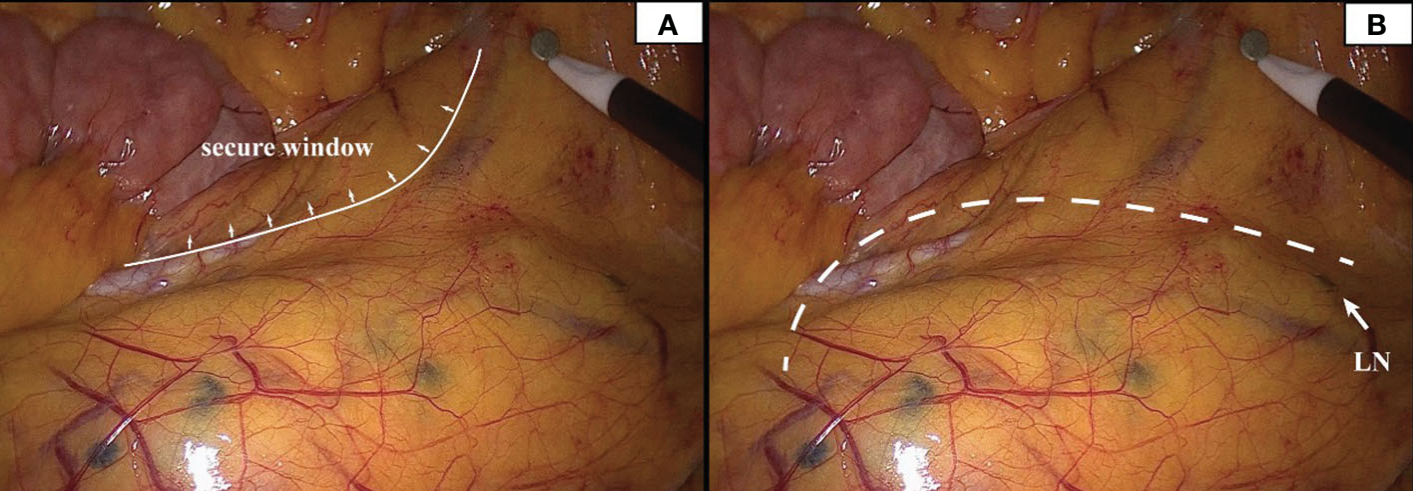
Deeply stained lymph nodes scattered in the mesentery can be seen in the presence of carbon nanoparticles. The inner boundary of the safety window can be seen in panel **(A)**. The white dotted line in panel **(B)** represents the surgical path, and the white arrow represents the deep-stained lymph node closest to the surgical path.

In this study, to ensure that the integrity of lymph node dissection is not compromised while protecting the marginal artery, 18 PCC patients underwent nano-carbon lymph node tracing (**Figure 8**). No deep-stained lymph nodes were detected outside the surgical path, signaling that the protection of the marginal arch did not impact lymph node dissection.

## Discussion

The sigmoid marginal arteries are located in the left colon mesentery, which supplies blood to the intestinal canal. They are emitted from IMA. Variations in IMA branch vessels are intricate. Previous studies have delineated the various types of IMA and its branches. These branches form arch bridges in pairs or independently supply the intestinal canal. In sigmoid or rectal cancer surgery, the marginal artery may be accidentally injured owing to the lack of laparoscopic perception. It is well established that high ligation of IMA, complete removal of the mesocolon, and central vascular ligation allow complete and maximum lymph node harvesting, resulting in lower local recurrence and superior overall survival rates (10). However, colonic ischemia may develop following the operation, which could be attributed to medical, pharmacological, or other surgical factors. Although this is not commonly encountered among skillful surgeons, its occurrence may have dire consequences. By accessing previous IMA angiograms from the Interventional Department, it was possible to detect marginal vessel weaknesses. In addition to the frequently mentioned Griffiths’ and Sudeck’s points, poor anastomosis of marginal arteries may also occur in other locations. Earlier studies have established that the blood perfusion at the proximal site of the anastomosis was markedly decreased by either IMA or LCA clamping (11, 12). Given this observation, some scholars recommend low ligation of IMA and the preservation of the LCA to maximize the marginal arterial blood supply; however, this procedure may result in lymphatic residue. In the present study, researchers hope to find a more efficient approach to visualize the marginal arteries to preserve blood supply and dissect the lymph nodes.

Many scholars are concerned about this issue, and studies have subsequently been carried out. Al-Asari SF (13) proposed the “critical zone” near IMV, which is bounded by the lower edge of the pancreas, the IMV, and the LCA, and emphasized the need to carefully separate the adjacent vessels of IMV during IMV ligation to avoid damaging the Drummond artery. However, ligating the IMV at the level of IMA root level and disconnecting the LCA jointly rarely injured the marginal blood supply in our center. In these patients, preoperative imaging and intraoperative monitoring exposed a vascular-free zone on the left side of LCA that is usually larger than 3 cm in diameter. Under the guidance of CME, the difficult-to-damage marginal arch of the descending colon was penetrated. It is more convenient to dissociate IMV and LCA, which is highly useful in guiding mesangial clipping and provides a priority breakthrough window for anatomical dissection of IMV and LCA. We were unable to find any articles discussing the same topic, yet it is present in numerous patients. This window was named the ‘Secure Window’ in the interim. As illustrated in **Figure 1**, the blue area is the secure window. Its upper edge is the descending branch of LCA, while the ascending branch of the first sigmoid artery forms its lower boundary. Its medial boundary is the IMV, while its lateral border is formed by the marginal artery. It may increase in size in patients with an unbroken marginal arch between the branches of LCA or SA. If d-LCA is missing and the anastomosis at the Griffiths’ point is weak, the LCA should be preserved. This concept is in line with the findings of Wang Y (14).

Unlike the loose vascular texture at the level of the IMA root, the vasculature near the sigmoid colon is intricate. There are abundant SA and anastomoses of the vascular arch. In this area, the secondary arch of the edge arch may co-exist, or it may be absent. When the edge arch anastomosis is adequate, the secondary arch is significantly weak. In contrast, when the edge arch anastomosis is weak, the secondary arch is compensated and is often more than 5 cm away from the intestinal wall. At this time, it is easy to damage the edge artery arch according to the 3 cm standard. This area was named ‘Dangerous Triangle’. In previous cases, there were 2 (2/183) cases of marginal artery injury during surgery, and more intestinal canals were detected and severed early under endoscopy. In radical sigmoid cancer surgery, branches of the sigmoid artery, the ascending SA, and descending SA can be visualized, and the triangle constructed from the two vessels with the marginal arch as the superior border was named ‘Dangerous Triangle’. In this region, poor anastomosis was identified in 5.2% of patients. When the marginal arterial anastomosis is weak in this region, the secondary arch/the tip of the triangle should be retained so that the proximal bowel receives a satisfactory blood supply. Similarly, there is also a triangle between a-LCA and d-LCA, but its relevance during sigmoid colon cancer surgery is negligible.

In our study, we proposed the concept of the secure window and the dangerous triangle based on the anatomy of the marginal vascular network in the mesocolon adjacent to the sigmoid colon. We recommend that the location of the secure window should be identified following high ligation of IMA. The surgeon needs to incise the left colon artery and IMV, then penetrate the secure window and dissect the mesentery within this area. When the sigmoid colon artery is visualized, edge anastomosis should be observed, and the secondary vascular arch should be retained if necessary to ensure sufficient intestinal blood supply. Meanwhile, the distance between the marginal vessels and the intestinal wall that could be damaged intraoperatively was measured. We postulate that it is safe and feasible to optimize the surgical concept based on the secure window and to exercise caution when encountering danger zones to avoid injury. This window is accountable for maximizing the extent of dissection as well as the marginal blood supply and minimizing unnecessary intraoperative injuries and the incidence of postoperative anastomotic leakage. Further prospective studies are warranted to validate the findings of our study.

Furthermore, we evaluated the anatomy of the vascular network as well as the anastomotic status based on preoperative imaging data. However, the marginal artery is very small and may be challenging to visualize with conventional imaging and limited technology.

The distance between the marginal artery and the sigmoid colon has been studied. Gao et al. (15) reported that the distance between the margin arch and the inner side of the colon was within 1cm for 90% (116/129), and over 1cm for 10% (13/129), among which the furthest distance was 3cm after autopsy. Gong et al. (16) conducted a gross anatomical study of 70 cases and found that the distance in the right and left-side colon was 2.5 ± 0.8cm and 4.2 ± 1.1cm respectively. Zhao et al. (17) performed autopsies on 50 children, and the results showed that the margin arterial arch was 1.2 ± 0.4cm away from the ascending colon, 2.2 ± 0.5cm from descending colon. The studies above were all based on autopsy. Due to long-term formalin immersion, the measured data of gross specimens were different from the morphology of living tissues, and the measured results were lack of clinical value. Yang et al. (18) did intraoperative anatomical observation on 105 patients and found that the margin artery was 0.6-5.9cm away from the colon wall, with a median of 0.8cm. However, the relationship between the marginal artery and the colonic wall is not two parallel lines. These studies did not mention the specific measurement plane and lack of detailed measurement methods, which was of little significance to actual surgery.

Our team noted that studies on this topic were scarce, and their parameters were outdated. Indocyanine green (ICG) fluorescent angiography is a method used to determine the presence of intestinal blood flow by using a near-infrared (NIR) camera to monitor real-time fluorescence during surgery following intravenous injection of ICG. Long-term usage of this method has corroborated its safety and efficacy, and it is extensively employed to evaluate cardiac output, liver blood flow, liver function, and retinal blood vessels. Meanwhile, the most prevalent application of indocyanine green is angiography during colorectal anastomosis (19, 20). For instance, Watanabe et al. (21) successfully assessed the blood flow of the colonic marginal artery near the sigmoid colon using the ICG fluorescence method. Likewise, Munechika et al. (22) observed the blood flow of the distal colon under fluorescence. In recent years, an increasing number of studies have utilized ICG fluorescence angiography to analyze blood perfusion and the formation of blood vessels. We firmly believe that intraoperative indocyanine green fluorescein angiography can help accurately portray the network of the colic marginal artery and reduce intraoperative marginal artery injury events, as demonstrated by our preliminary experiment.

Indocyanine green (ICG) is an intravenous drug that can rapidly bind to plasma proteins and send infrared signals under *in-situ* laser excitation. Kudszus (23) observed tissue perfusion using ICG fluorescence angiography. This retrospective study uncovered that reoperation of the anastomotic leakage was lowered by 60% when visualization was performed. Mehraneh (24) performed an identical experiment and reported that the use of ICG fluorescence development in robotic surgery could decrease the incidence of anastomotic leakage to 6% (vs. 18% in the control group). This approach was also employed herein, but we discovered that patients with high BMI suffered from mesangial hypertrophy, and ICG was difficult to develop marginal vessels. Considering the above findings, the anastomosis of the marginal arch was observed, and the distance between the primary/secondary arch and the intestinal wall was measured to provide a broad understanding of the location of the vessel’s course during surgery, which is crucial for avoiding vascular injury.

Vascular variations in the left colon are complex; even with adequate preparation, it is still possible to injure the marginal artery. Indeed, there were two patients with intestinal malrotation in our study. Its detection rate is reported to be between 1.9% and 2.4% (25). The IMA of such patients is distinct from healthy individuals, forming a ‘bear claw’ structure. Patients with intestinal malrotation usually have a shorter LCA which directly forms the MA. This indicates that they lack a secure window and that surgeons must disconnect the left colonic artery before bifurcation.

In the last phase of our study, using the nano-carbon tracer imaging, the preservation of secondary vascular arch while taking into account the complete lymph node dissection. However, we also found that the farthest distance of the secondary arch was 10 cm (2/96, 2.1%) from the intestinal wall. Besides, the secondary arch was close to the IMA trunk. If the marginal arch is weak and the secondary arch is distant, we recommend clipping the mesentery with the support of vascular imaging during the operation.

## Conclusion

It is reasonable and feasible to perform preoperative imaging evaluation, intraoperative ICG fluorescence imaging, and surgical pathway optimization based on the concept of the secure window and dangerous triangle during the perioperative period for PCC surgery. Caution is warranted when locating the marginal artery in potentially dangerous areas to avoid injury, which maximizes the extent of dissection and marginal blood supply and minimizes the incidence of unnecessary intraoperative injury and postoperative bowel ischemia. Further prospective studies are warranted to validate the findings of this study.

## Data availability statement

The raw data supporting the conclusions of this article will be made available by the authors, without undue reservation.

## Ethics statement

The studies involving humans were approved by ethics committee of Guangdong Provincial Hospital of Chinese Medicine. The studies were conducted in accordance with the local legislation and institutional requirements. The participants provided their written informed consent to participate in this study. Written informed consent was obtained from the individual(s) for the publication of any potentially identifiable images or data included in this article.

## Author contributions

JW: Data curation, Methodology, Resources, Writing – original draft, Writing – review & editing. JX: Resources, Software, Writing – review & editing. XL: Data curation, Methodology, Writing – review & editing. JL: Methodology, Resources, Writing – review & editing. WLL: Data curation, Formal Analysis, Methodology, Resources, Writing – review & editing. XY: Data curation, Formal Analysis, Writing – review & editing. XF: Data curation, Formal Analysis, Methodology, Writing – review & editing. BZ: Formal Analysis, Software, Writing – review & editing. WJL: Formal Analysis, Investigation, Writing – review & editing. XT: Resources, Software, Writing – review & editing. LA: Formal Analysis, Investigation, Writing – review & editing. ZC: Data curation, Resources, Writing – review & editing. HL: Methodology, Supervision, Validation, Visualization, Writing – original draft. DD: Methodology, Supervision, Validation, Visualization, Writing – original draft.
